# Supplementary material for: Initial Healing Effects of Platelet-Rich Plasma (PRP) Gel and Platelet-Rich Fibrin (PRF) in the Deep Corneal Wound in Rabbits
Source: Bioengineering (Basel). 2022 Aug 20;9(8):405. doi: 10.3390/bioengineering9080405 (PMC9405118; doi:10.3390/bioengineering9080405)
Supplement: Supplementary file 1 [file bioengineering-09-00405-s001.zip › bioengineering-1842353-supplementary.pdf]

Supplementary Table S1. Modified MacDonald-Shadduck scoring system.

**Corneal Opacity**	
0	Normal cornea. Appears with the slit lamp as having a bright grey line on the epithelial surface and a bright grey line on the endothelial surface, with a marblelike grey appearance of the stroma.
1	Some loss of transparency. Only the anterior half of the stroma is involved as observed with an optical section of the slit lamp. The underlying structures are clearly visible with diffuse illumination, although some cloudiness can be readily apparent with diffuse illumination.
2	Moderate loss of transparency. In addition to involving the anterior stroma, the cloudiness extends all the way to the endothelium. The stroma has lost its marblelike appearance and is homogeneously white. With diffuse illumination, underlying structures are clearly visible.
3	Involvement of the entire thickness of the stroma. With optical section, the endothelial surface is still visible. However, with diffuse illumination, the underlying structures are just barely visible (to the extent the observer is still able to grade flare, iritis, and note lenticular changes).
4	Involvement on the entire thickness of the stroma. With the optical section, cannot clearly visualize the endothelium. With diffuse illumination, the underlying structures cannot be seen. Cloudiness removes the capability for judging and grading aqueous flare, iritis, and lenticular changes. Can see posterior margin of the cornea, but cannot score by slit lamp examination.
5	Cannot see posterior margin of cornea.
6	Corneal perforation.

Supplementary Table S2. The number of samples in each group.

	1W	2W	3W	4W	6W	8W
**Group1**	8	8	8	4	4	4
**Group2**	8	8	8	4	4	4
**Group3**	9	9	9	4	4	4
**Group4**	8	8	8	4	3	3

Supplementary Table S3. Rate of hypopion as a complication, % (affected eyes/total number).

	1W	2W	3W	4W	6W	8W
**Group1**	0(0/8)	0(0/8)	0(0/8)	0(0/4)	0(0/4)	0(0/4)
**Group2**	37.5(3/8)	37.5(3/8)	0(0/8)	0(0/4)	0(0/4)	0(0/4)
**Group3**	44.4(4/9)	22.2(2/9)	0(0/9)	0(0/4)	0(0/4)	0(0/4)
**Group4**	62.5(5/8)	50(4/8)	0(0/8)	0(0/4)	0(0/3)	0(0/3)

Supplementary Table S4. Rate of corneal stromal inflammation and ulcer as a complication, % (affected eyes/total number).

	**1W**	**2W**	**3W**	**4W**	**6W**	**8W**
**Group1**	0(0/8)	0(0/8)	0(0/8)	0(0/4)	0(0/4)	0(0/4)
**Group2**	0(0/8)	12.5(1/8)	12.5(1/8)	25(1/4)	0(0/4)	0(0/4)
**Group3**	0(0/9)	0(0/9)	22.2(2/9)	0(0/4)	0(0/4)	0(0/4)
**Group4**	0(0/8)	0(0/8)	75(6/8)	75(3/4)	0(0/3)	0(0/3)

Supplementary Table S5. Rate of corneal perforation as a complication, % (affected eyes/total number).

	1W	2W	3W	4W	6W	8W
**Group1**	0(0/8)	0(0/8)	0(0/8)	0(0/4)	0(0/4)	0(0/4)
**Group2**	0(0/8)	0(0/8)	0(0/8)	0(1/4)	0(0/4)	0(0/4)
**Group3**	0(0/9)	0(0/9)	0(0/9)	0(0/4)	0(0/4)	0(0/4)
**Group4**	0(0/8)	0(0/8)	12.5(1/8)	25(1/4)	0(0/3)	0(0/3)
